# Comprehensive Study of Multiple Stages Progressing to Nonalcoholic Steatohepatitis with Subsequent Fibrosis in SD Rats

**DOI:** 10.3390/ijms18081681

**Published:** 2017-08-18

**Authors:** Lulu Wang, Susu Wu, Minxuan Cai, Ji Ma, Shengcun Li, Maoru Li, Yan Xu, Lixin Wei, Jing Shang

**Affiliations:** 1 State Key Laboratory of Natural Medicines, China Pharmaceutical University, Nanjing 210009, China; wuyuzhilu@sina.com (L.W.); cpu920415@163.com (S.W.); cmxc3h6@126.com (M.C.); Matthewmj@yeah.net (J.M.); ShengcunL@163.com (S.L.); 18851722588@163.com (M.L.); 18260090078@163.com (Y.X.); 2 Jiangsu Key Laboratory of TCM Evaluation and Translational Research, China Pharmaceutical University, Nanjing 211198, China; 3 Key Laboratory of Tibetan Medicine Research, Northwest Institute of Plateau Biology, Chinese Academy of Sciences, Xining 810008, China; Lxwei@nwipb.cas.cn; 4 Qinghai Key Laboratory of Tibetan Medicine Pharmacology and Safety Evaluation, Northwest Institute of Plateau Biology, Chinese Academy of Sciences, Xining 810008, China

**Keywords:** nonalcoholic steatohepatitis, liver fibrosis, lipid metabolism, chronic inflammation, oxidative stress, insulin resistance

## Abstract

Because of the absence of the time course of histological nonalcoholic fatty hepatitis with subsequent fibrotic progression, the effective approaches available for controlling the onset and progression of non-alcoholic steatohepatitis (NASH) remain limited. Therefore, we detected the serum and liver tissue related lipid metabolism disorder, liver pathology and relative molecular makers alteration dynamically in a high fat-sucrose diet during different time points. High fat-sucrose diet significantly increased the serum lipid level on day 10. The excess lipid accumulation in liver was referred to as simple steatosis after the feeding of a high fat-sucrose diet for 20 days. The high fat-sucrose diet induced a hepatic inflammation response on day 30. Similarly, hepatic fibrosis was also initiated on day 30 and gradually formed from the 30th to the 50th day. Oxidative stress may be related with the process from NASH to liver fibrosis. Insulin resistance was involved in the progression from hepatic steatosis to NASH with hepatic fibrosis from the 20th to the 50th day. In conclusion, we established a high fat-sucrose diet induced nonalcoholic fatty hepatitis with liver fibrosis rat model, which presented the time course of histological nonalcoholic steatohepatitis and the initiation and progression change of characteristic molecular makers in the process from steatosis to hepatic fibrosis.

## 1. Introduction

Non-alcoholic steatohepatitis (NASH), a subtype of the spectrum of non-alcoholic fatty liver diseases (NAFLD), is the most common condition of chronic liver disease worldwide, representing the hepatic manifestation of the metabolic syndrome [1]. In addition, its histological characteristics are steatosis combined with hepatic inflammation and hepatocellular injury [2]. NASH is the progressive consequence of excess lipid accumulation in liver referred as steatosis or non-alcoholic fatty liver (NAFL) that continues to the progression of hepatic fibrosis or even cirrhosis and hepatocellular carcinoma [3]. The prevalence of the disease has been dramatically increasing in western countries among obese and non-obese people and recently in Asia [4,5]. Since the initiation and progression of NAFLD/NASH is a slow and progressive process, applying an appropriate therapy that can target most of its crucial pathological events (e.g., insulin resistance, lipid accumulation, inflammation and fibrosis), should theoretically prevent or reverse the process of NASH to a nearly healthy condition [6,7].

Hepatic steatosis is recognised as a prerequisite for NASH and has also been identified as an independent risk factor for hepatic fibrosis [2]. Indeed, the strongest predictor of fibrosis progression in NAFLD is the presence of steatohepatitis [8]. But most of the current studies focus on the special fatty liver pathological state as NAFL, NASH, hepatic fibrosis [9,10,11]. It is known that in the NAFL stage, an accumulation of intrahepatic triglyceride is the hallmark feature of NAFL, and lipid synthesis was recognized as the important factor in fat accumulation in liver [12]. Peroxisome proliferator-activated receptor γ2 (*PPARγ2*), sterol regulatory element-binding protein-1c (*SREBP-1c*) and its downstream target gene fatty acid synthase (*FAS*) are the good markers of the presence and the degree of hepatic steatosis [13,14]. With respect to the NASH stage, activated Kupffer cell, hepatic inflammation promoters, such as tumor necrosis factor-α (*TNF-α*), interleukin-6 (*IL-6*), monocyte chemotactic protein-1 (*MCP-1*) gene expression are the important features of the progression from NAFL to NASH [15]. Finally, the obvious characteristics of the progression of NASH to fibrosis are hepatic stellate cell activation and relatively high expression of fibrotic genes, such as transforming growth factor-β (*TGF-β*), α-smooth muscle actin (*α-SMA*), and matrix metalloproteinase-13 (*MMP-13*) [16]. In addition, insulin resistance and oxidative stress are also involved in the development and progression of NASH [17]. To date, a few studies have reported the simple hepatic lipid status of the steatotic, NASH, and fibrotic liver stage [18]. However, few reports comprehensively show serum or liver lipid metabolism, inflammation changes, and liver fibrosis in the whole stage proceeding from steatosis to hepatic fibrosis.

Up to date, dietary-induced models of NASH with fibrosis have contained genetic manipulation, forced overfeeding or contrived diets that are deficient in methionine and choline [19]. Although each of these models has been valuable, they might not have presented the etiology and whole-body/liver pathology of human NASH. NASH is not only a progressive stage, but also an available intervention phase in clinics. Indeed, the investigation of the pathogenic or therapeutic factors involved in NASH has been hampered by the lack of an available experimental model. Here, we aimed to show a dietary model of NASH with liver fibrosis on the whole-body/liver pathology in a relatively short period and dynamically clarified the relative changes of lipids, inflammation, and fibrosis. Having an available NASH model would be helpful in developing new therapeutic strategies and evaluating the effects of potential drugs in vivo.

## 2. Results

### 2.1. Effect of a High Fat-Sucrose Diet on Metabolic Profiles in Sprague-Dawley (SD) Rats

The body weight of the model group was obviously less than the corresponding control group from the 10th day to 50th day. Conversely, the liver index of the model group was significantly higher from day 10 to day 50. The food intake of SD rats was similar among the groups from day 10 to day 50 (Table 1).

**Figure 1:**
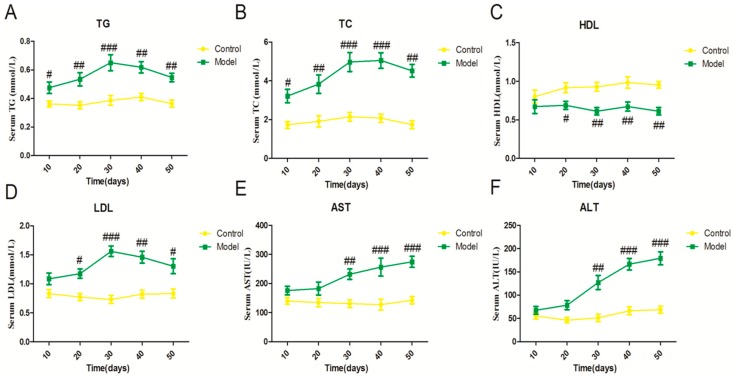
Effect of a high fat-sucrose diet on serum lipid profiles and liver function indicators. The serum lipid parameters of sprague-dawley (SD) rats induced by a high fat-sucrose diet on day 10, 20, 30, 40, 50. (**A**) Total triglyceride (TG); (**B**) Total cholesterol (TC); (**C**) High-density lipoprotein (HDL); (**D**) Low-density lipoprotein (LDL); (**E**) Aspartate aminotransferase (AST); (**F**) Alanine aminotransferase (ALT). The values are shown as the means ± standard error of mean (SEM) (*n* = 6). Compared with the corresponding control group, ^#^ *p*＜ 0.05, ^##^ *p* < 0.01, ^###^ *p* < 0.001.

As our result show, the serum triglyceride (TG) concentration gradually elevated in the model group compared with the corresponding control group from day 10 to day 30, and the model group was significantly lower from 40 days (Figure 1A). Consistent with the serum TG change, serum total cholestrol (TC) and low density lipoprotein (LDL) exhibited a similar tendency (Figure 1B–D). These data suggest that the serum lipid disorder was initiated from day 10 to day 50 induced by a high fat-sucrose diet. In addition, serum alanine aminotransferase (ALT) and aspartate transaminase (AST) levels of rats exhibited a sharp difference in the model group compared with the control group on day 30, which showed hepatic injury from day 30 (Figure 1E,F).

### 2.2. Effect of a High Fat-Sucrose Diet on Hepatic Lipid Accumulation

**Figure 2:**
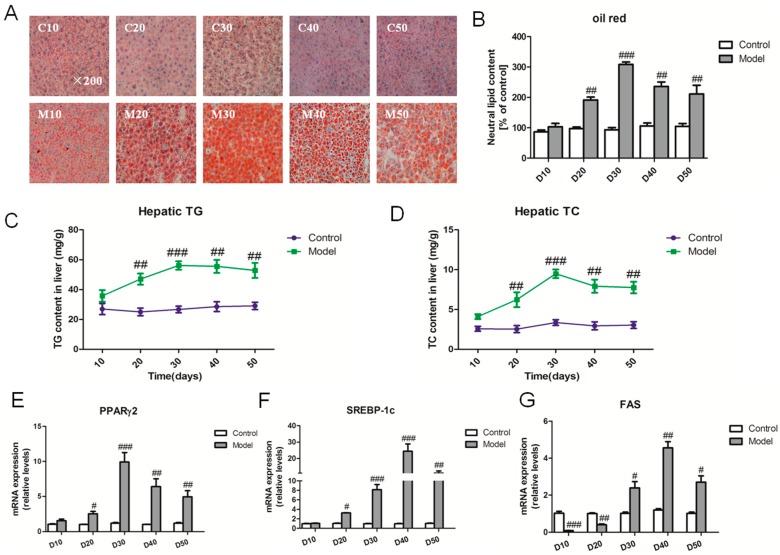
High fat-sucrose diet induced fat overload in liver. The hepatic lipid metabolism of SD rats induced by a high fat-sucrose diet on day 10, 20, 30, 40, 50. (**A**) Oil red O staining (small red circles) shows neutral lipid deposition in the liver (original magnification 200×). Small red circles indicate the formation of large cytoplasmic lipid droplets; (**B**) Semi-quantitative analysis of the contents of lipid droplets; (**C**) Hepatic TG content; (**D**) Hepatic TC content; the relative lipid synthesis gene *PPARγ2* (**E**), *SREBP-1c* (**F**), *FAS* (**G**) mRNA expression. The values are shown as the means ± SEM (*n* = 6). Compared with the corresponding control group, ^#^ *p* < 0.05, *^##^ p* < 0.01, *^###^ p* < 0.001.

To investigate hepatic lipid metabolism induced by a high fat-sucrose diet, we examined lipid droplet changes during the period of 50 days. Under the high fat-sucrose diet, the lipid droplet content exhibited an increasing trend without any significant difference compared with the control group on the 10th day, whereas the hepatic lipid droplet content increased higher than the relative control group from the 10th to the 50th day (Figure 2A,B). Consistent with the lipid droplet change, the hepatic TG and TC contents differed significantly between the model group and corresponding control group from day 20 to day 50 (Figure 2C,D). To further explore the molecular process underlying the lipid synthesis, we surprisingly found that the lipogenic genes (*PPARγ2*, *SREBP-1c*, *FAS*) were strikingly up-regulated from day 20 to day 40 but down-regulated on the 50th day (Figure 2E–G). In order to further the finding, we also examined the relative hepatic lipid metabolism factors (Figure S1).

### 2.3. Effect of the High Fat-Sucrose Diet on the Hepatic Inflammation Response

**Figure 3:**
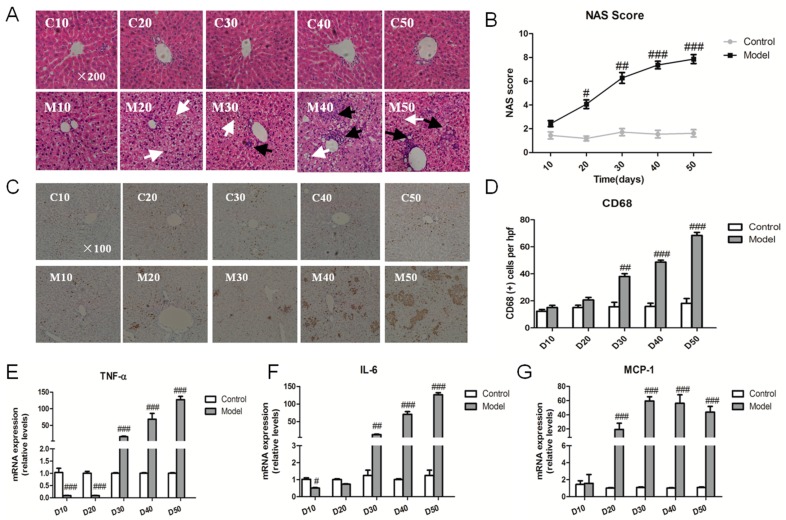
High fat-sucrose diet promoted inflammation development in the liver. The hepatic inflammation change of SD rats induced by a high fat-sucrose diet on day 10, 20, 30, 40, 50. (**A**) Typical hematoxylin–eosin (HE) staining results of each group of rats (×200). White and black arrows display fat vacuole of hepatocytes and infiltration of inflammatory cells, respectively; (**B**) NAFLD activity score (NAS) of each group of SD rats after Non-alcoholic steatohepatitis (NASH) induction; (**C**) CD68 positive Kupffer cell (×100); (**D**) Semi-quantitative analysis of the CD68 positive Kupffer cell. The relative inflammation factors of the gene *TNF-α* (**E**), *IL-6* (**F**), *MCP-1* (**G**) mRNA expression. The values are shown as the means ± SEM (*n* = 6). Compared with the corresponding control group, ^#^ *p* < 0.05, *^##^ p* < 0.01, *^###^ p* < 0.001.

To determine the hepatic injury process from NAFL to NASH, we detected hepatic histopathological change by typical hematoxylin-eosin (HE) staining (Figure 3A). NAFLD activity score (NAS) analysis indicated that the rats fed high fat-sucrose diet presented notable steatosis, hepatocellular ballooning and inflammatory cell infiltration (Figure 3B). Circulating inflammation factors (TNF-α, IL-6, IL-1β) were also notably changed in the model group on day 30 (Figure S1). Moreover, the number of CD68 positive making Kupffer cells significantly increased compared with the relative control group since the 30th day (Figure 3C,D), and the myeloperoxidase (*MPO*) mRNA level was up-regulated (Figure S2D), which suggested a hepatic inflammation response originated from day 30. In addition, the important inflammation factor *TNF-α* and *IL-6* mRNA expression were striking up-regulated from day 30 compared with the corresponding control group, except for the *MCP-1* gene from day 20 (Figure 3E–G). To further confirm the hepatic inflammation response, we detected the change in serum inflammation factors and the expression of hepatic inflammation genes (Figure S2).

### 2.4. Effect of the High Fat-Sucrose Diet on Hepatic Fibrosis

**Figure 4:**
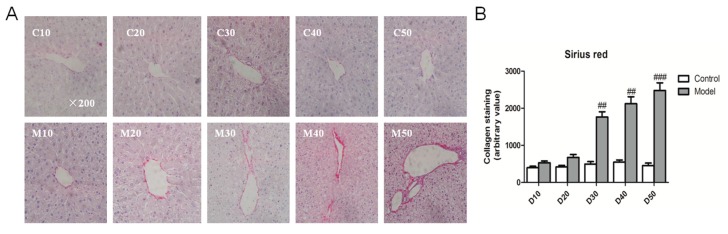

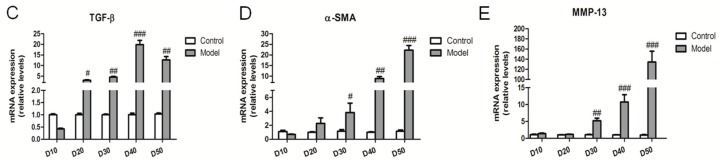
High fat-sucrose diet caused hepatic fibrosis in NASH rats. The hepatic fibrosis of SD rats fed a high fat-sucrose diet on day 10, 20, 30, 40, 50. (**A**) Sirius red (SR) staining; (**B**) Semi-quantitative analysis of the SR staining of every group. The relative fibrosis gene *TGF-β* (**C**), *α-SMA* (**D**), *MMP-13* (**E**) mRNA expression. The values are expressed as the means ± SEM (*n* = 6). Compared with the corresponding control group, ^#^ *p* < 0.05, ^##^ *p* < 0.01, *^###^ p* < 0.001.

To clarify NASH progression from NASH to hepatic fibrosis, we used sirius red (SR) staining to indirectly reflect activated stellate cells in liver. Sirius red staining represented that the hepatic collagen formation of NASH rats was strikingly higher than that of the corresponding control rats from the 30th day (Figure 4A,B). The gene expression of both *α-SMA* and *MMP-13* showed an apparent up-regulation in NASH rats compared with control rats from the 30th day; contrasting results were observed for *TGF-β* from the 20th day. Additionally, the fibrotic genes exhibited a similar trend for promoting fibrosis of NASH rats over 30 days (Figure 4C–E).

### 2.5. Effect of the High Fat-Sucrose Diet on the Hepatic Oxidative Stress Response

**Figure 5:**
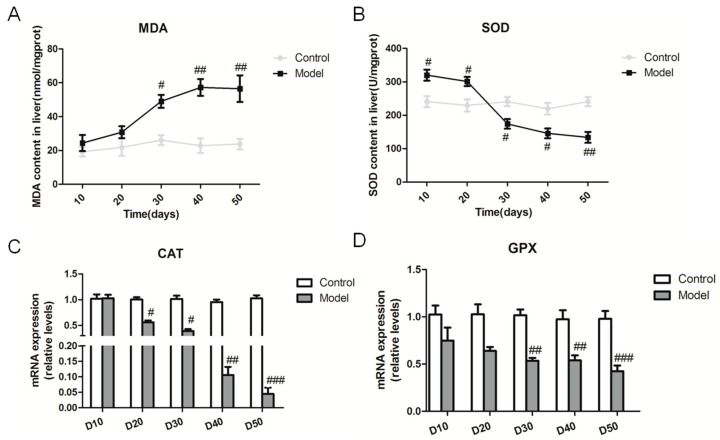
High fat-sucrose diet triggered hepatic oxidative stress in NASH rats. The hepatic oxidative stress of SD rats induced by the high fat-sucrose diet on day 10, 20, 30, 40, 50. (**A**) Malonaldehyde MDA) content; (**B**) Superoxide dismutase (SOD) content. The relative oxidative stress gene expression of *CAT* (**C**), *GPX* (**D**). The values are expressed as the means ± SEM (*n* = 6). Compared with the corresponding control group, ^#^ *p* < 0.05, ^##^ *p* < 0.01, *^###^ p* < 0.001.

It is known that oxidative stress plays a pivotal part in the development and progression of NASH. We measured the malonaldehyde (MDA) and superoxide dismutase (SOD) contents in the liver of SD rats fed the high fat-sucrose diet (Figure 5A,B). The hepatic MDA content exhibited a gradually increasing tendency in general during the experimental period. Moreover, the hepatic MDA content was significantly higher compared with the control group on day 30, and the trend goes to a plateau phase after the 30th day opposed with the hepatic SOD content. Catalase (*CAT*) and glutathione peroxidase (*GPX*) gene mRNA expression were down-regulated obviously compared with the relative control group from day 10 to day 50, which suggested the high fat-sucrose diet decreased the hepatic antioxidant capacity (Figure 5C,D).

### 2.6. Effect of the High Fat-Sucrose Diet on Hepatic Insulin Resistance

**Figure 6:**
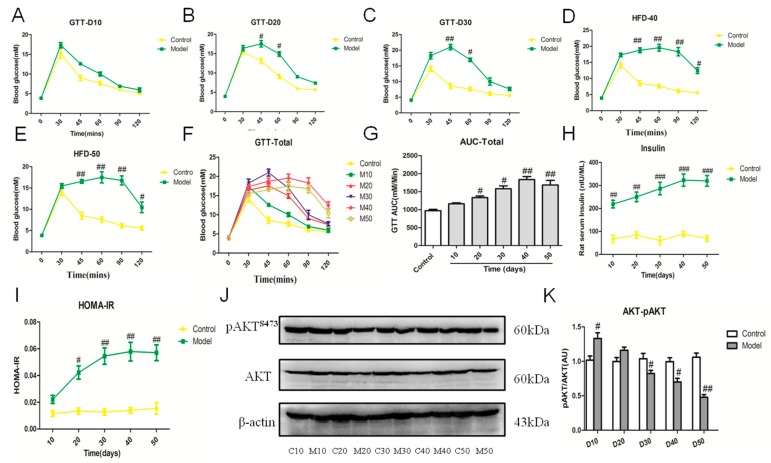
High fat-sucrose diet accelerated insulin resistance in NASH rats. The hepatic insulin resistant of SD rats induced by the high fat-sucrose diet on day 10, 20, 30, 40, 50. The blood glucose concentration of day 10 (**A**); day 20 (**B**); day 30 (**C**); day 40 (**D**); day 50 (**E**); total period (F); Area under the cure (AUC) of total (**G**). Serum insulin content (**H**). Homeostasis model assessment index of insulin resistance (HOMA-IR) (**I**). The protein expression of phospho-AKT (Ser473) (pAKT^ser473^) and protein kinase B (AKT) (**J**). Semi-quantitative of pAKT^ser473^ and AKT protein expression (**K**). The values are expressed as the means ± SEM (*n* = 6). Compared with the corresponding control group, ^#^ *p* < 0.05, ^##^ *p* < 0.01, ^###^ *p* < 0.001.

As insulin resistance plays a critical role in development and progression of NAFLD, we checked the insulin sensitivity of rats induced by the high fat-sucrose diet. Our result showed that the blood glucose level of fasting rats was apparently higher than that of the control group from day 10 to day 50, and the area under the cure (AUC) of the glucose tolerance test (GTT) exhibited an increasing tendency during the experimental period (Figure 6A–E). Insulin resistance was measured by applying the homeostasis model assessment index of insulin resistance (HOMA-IR) [20]. The insulin level and HOMA-IR exhibited similar trends, which verified insulin resistance initiated on day 20 in rats fed the high fat-sucrose diet (Figure 6F,G). The ratio of phospho-AKT (Ser473) (pAKT^ser473^) and protein kinase B (AKT), which is the marker of insulin resistance, also demonstrated the decreasing insulin sensitivity from day 30 (Figure 6H,I).

## 3. Discussion

The pathogenic process of NAFLD is strongly linked to over-nutrition. Meanwhile, lipid accumulation, insulin resistant, oxidative stress and inflammation response have been widely suggested to play a pivotal role in the transition from steatosis to NASH [21,22]. Our finding indicated a NASH model progression with obvious serum lipid disorder and hepatic lipid dysregulation, which is considered as NAFL, and the induction of insulin resistance after 20 days of a high fat-sucrose diet. Fat overload from liver-induced oxidative stress led to liver inflammation abnormalities and liver injury in the period of 30 days in rats fed the high fat and sucrose diet. Furthermore, the high fat and sucrose diet promoted NASH to transition to liver fibrosis after 50 days. Consistent with lipid metabolism disorders, insulin resistance, inflammation, and oxidative stress involved in NAFLD are still potential therapeutic targets for NAFLD [23].

In the present study, we evaluated the lipid profiles of rats at different times of NASH progression. In particular, the high fat-sucrose diet dramatically increased the serum lipid content and lipid accumulation in the liver after 20 days, while increases in the serum TC and TG contents were initiated on the 10th day. To further investigate the underlying mechanisms of overload fat in the liver, the expressional changes of lipogenic genes (*PPARγ2*, *SREBP-1c* and *FAS*) were measured, as well as the upstream gene of hepatic lipid metabolism (Figure S1). This result showed upregulated *PPARγ2*, *SREBP-1c* and *FAS* genes expression in a time-dependent manner except for day 50, which indicated a downward trend. Therefore, we speculated that enhanced hepatic lipogenesis may lead to increasing TG, TC, and LDL contents in the serum of rats, and hepatic inflammation or fibrosis may cause a decreased hepatic lipid content [24]. This is in line with hepatic steatosis as a marker of metabolic dysfunction [25]. This dynamic lipid disorder suggests that the system lipid content should be improved for treatment of NAFL, and anti-inflammation is a preventive treatment strategy [26].

The main pathological difference between hepatic steatosis and NASH is the occurrence of hepatic inflammation. The inflammation response has been involved in the pathogenesis of NASH. Macrophages and monocyte populations are a major source of proinflammation cytokines in liver and are the key players in NASH progression and treatment [27]. Our finding also indicated that the increased inflammation factor in serum, enhanced recruitment of Kupffer cells and upregulated inflammation factors were time-dependent, such as TNF-α, IL-1β, IL-6. In humans, the NAS and fibrosis stages are used for the pathologic diagnosis of NASH [28]. We evaluated the NAS score and fibrosis stage using HE and SR staining in liver tissues. Indeed, our results showed the gene expression of *TNF-α*, *IL-6*, *MCP-1* was upregulated from day 30, induced by the high fat-sucrose diet. In accordance with our results, the blocking inflammation response may be the key treatment for NASH rather than decreasing the hepatic lipid content [7,8,9]. Lipid peroxidation and triggered oxidative stress are common consequences of NAFL and insulin resistance during the progression of NASH [29], consistent with our result that raising the MDA content and decreasing SOD in the liver of rats fed the high fat-sucrose diet on day 30. Moreover, *CAT* and *GPX* gene expression, which reflects the extent of intrinsic antioxidation capacity, was downregulated. In addition, more than 30 days of the high fat-sucrose diet gradually induced liver fibrosis, which represents NASH progression to fibrosis. According to our above results, we suggest that an anti-inflammatory combined anti-oxidant approach may be a more effective strategy for NASH development and progression [15,29].

Our finding showed obviously that different cells are involved in the progression of NASH. In the NAFL period, the high-fat sucrose diet induced fat overload in the liver and recruited Kupffer cells, which initiated the inflammation response. Furthermore, lipid accumulation and hepatic inflammation activated stellate cells during NASH progression to liver fibrosis. Hepatocytes are an important location for lipid metabolism. FFA overload triggers steatosis in hepatocytes. Due to a high hepatic lipid content initiating hepatocyte injury, recruitment of Kupffer cells occurred and furthermore, triggered NASH development [30,31]. Similar to our results, the number of CD68-positive Kupffer cells was significantly higher than in the control group during the NASH period from the 30th to 50th day. Activated hepatic stellate cells generate an important source of collagen and other extracellular matrix proteins in liver, which is the hallmark of liver fibrosis [32]. According to our present study, activated stellate cells stained with sirius red suggested collagen production in NASH with fibrosis rats was apparently higher than in the NASH rats fed the high fat-sucrose diet over 30 days. Moreover, the increasing expression of relative genes with liver fibrosis (*TGF-β*, *α-SMA*, *MMP-13*) also demonstrated hepatic fibrosis initiation on the 30th day of excessive nutrition intake and gradually aggravated the severity of liver fibrosis from the 30th to 50th day. This result suggested that blocking fibrosis should start an intervention and treatment on day 30. Insulin resistance and oxidative stress play important roles in the development and progression of NAFLD [33]. In our study, insulin resistance was implicated in the development and progression of NASH since the 20th day. Moreover, understanding the mechanisms of oxidative stress in the pathogenesis of NASH is of benefit for developing a targeted therapy [15]. Therefore, insulin resistance and oxidative stress may be two potential therapeutic targets in non-alcoholic steatohepatitis [34]. While the ideal treatments for NAFLD should reverse the accumulation of triglycerides in hepatocytes and effectively suppress hepatic inflammation, which may prevent simple steatosis from developing into NASH and fibrosis [26]. Our result showed the treatment approach should depend on the severity of the specific fatty liver condition.

This model shows the histopathological progression that is very similar to the human NAFLD, especially the rapid and gradual transition from steatosis to NASH with fibrosis. These changes further progressed to subsequent fibrosis and were accompanied with insulin resistance, which may worsen lipogenesis in liver and promote the progression to steatohepatitis and even fibrosis. Thus, we claimed that fatty liver induced by feeding of the high fat-sucrose diet with propylthiouracil exhibits the histopathological characteristic of NASH in humans with both steatohepatitis and outstanding insulin resistance. This is in agreement with adding 0.25% propylthiouracil to the diet, which might have contributed to the development of pathological features of NASH and systemic IR [35]. Insulin resistance is partly caused by the absence of insulin receptor signaling, such as down-regulated phosphorylation of AKT protein [36]. But our finding showed increased insulin sensitivity after 10 days of the high fat-sucrose diet, opposite to insulin resistance of rats after 20 days under a high fat-sucrose diet. In accordance with the compensatory mechanism for the initial accumulation of fat in a diet-induced NAFLD mode [37], we presumed that the early compensatory mechanism of the liver might be involved in insulin sensitivity. Consistent with our finding, insulin resistance has a crucial role in the initiation and progression of NASH towards fibrosis [38]. Although one limitation is associated weight loss, the model is at least equivalent, if not better than the existing NASH models, such as those induced by high fat/calorie (HFC) or a methionine and choline deficient diet (MCD). The further application of this model is an available tool to investigate the NASH pathophysiology and evaluate the possible therapeutic strategies.

In summary, we demonstrated that a high fat-sucrose diet induced serum lipid disorder and promoted NAFL progression to NASH and to liver fibrosis in an SD rat model likely through initiating insulin resistance and oxidative stress. According to hepatic injury and fibrosis and apparent insulin resistance, the present study demonstrated the similarity of a rat NASH model to human disease [21]. Our findings lay the groundwork for a treatment strategy for NAFL and NASH with liver fibrosis and explore the potential mechanism for NASH progression, as well as potential drugs for screening. This model is particularly useful for further exploration of the crucial role of insulin resistance in the initiation and progression of NASH to hepatic fibrosis.

## 4. Materials and Methods

### 4.1. Animal Studies

Forty-eight male Sprague-Dawley (SD) rats weighing 180~220 g at 5–6 weeks of age were purchased from the Shanghai Super-B&K laboratory animal Corp. Ltd. (Shanghai, China). All SD rats were provided free access to food and water during the acclimatization and experimental periods. All SD rats were exposed to 12 h of light/dark during the experiment. This study was approved by the Science and Technology Department of Jiangsu Province (SYXK(SU)2016-0011). All animal experiments followed the standard ethical guidelines under the ethical committees mentioned above. After acclimatization for one week, the SD rats were randomly divided into eight experimental groups (*n* = 6) as follows: (a) Control group: fed with a basal diet (360 kcal/100 g, 13.3 g/100 g from fat, 26.2 g/100 g from protein, and 60.5 g/100 g from carbohydrate); (b) Model group: fed with a high fat-sucrose diet (506.8 kcal/100 g, lard, 10 g/100 g; cholesterol, 2 g/100 g; egg yolk power, 5 g/100 g; sucrose, 10 g/100 g; propylthiouracil, 2 g/100 g; basal diet, 72.8g/100 g). The basal diet and high fat-sucrose diet were provided by the Jiangsu Xietong Medical and Biological Corporation (Nanjing, China); during the experiment period, food intake and the body weight were recorded daily. Six rats from each group were harvested on day 10, 20, 30, 40, 50. At the end of each period, all SD rats were fasted for 12 h and sacrificed under anesthesia for collection of blood and liver.

### 4.2. Serum Biochemical Analysis

After finishing the study, all SD rats were sacrificed at once, and serum was collected instantaneously by centrifugation at 1200× *g* for 15 min. The serum lipid index: total triglycerides (TG), total cholesterol (TC), low-density lipoprotein (LDL), and high-density lipoprotein (HDL) were evaluated the lipid changes. Aspartate and alanine transaminases (AST and ALT) were examined to assess the hepatic injury. All above-mentioned indexes were measured by commercially available kits (Nanjing Jiancheng Bioengineering Institute, Nanjing, China). Enzyme-linked immunoassay (ELISA) measurements of serum TNF-α, IL-1β and IL-6 were performed following the user instructions (Neobioscience Technology Co., Ltd., Shenzhen, China). An insulin ELISA kit was purchased from Cusabio (Cusabio Biotech, Wuhan, China).

### 4.3. Liver Histopathological Analysis

Histopathological research was conducted on standardized specimens from specified portions of liver. Briefly, liver tissues were fixed in 4% paraformaldehyde for 4 hours and dehydrated in a series of ethanol and embedded in paraffin wax. Sections (4-mm-thick) were stained with hematoxylin-eosin (HE) staing before being analyzed under an Olympus biological microscope (BX53, Tokyo, Japan). The NAFLD activity score (NAS) of each group was calculated as previously described [28]. Oil Red O-stained lipid droplets of the fresh liver samples were analyzed to quantify lipid content by cell imaging under an Olympus-BX53 biological microscope. Areas of stained droplets were determined using Image J software and normalized to the areas of rat hepatocytes. Sirius red and CD68 (1:800, ab31630, abcam, Cambridge, UK) were used for histological analysis under a light microscope Olympus-BX53.

### 4.4. Quantitative Real-Time Polymerase Chain Reaction (RT-qPCR)

Total RNA was extracted from liver using TRIZOL reagent (Invitrogen, Carlsbad, CA, USA), and cDNA was synthesized with PrimeScript™ RT Master Mix (Takara, Japan) according to the manufacturer’s protocols. Quantification of gene expression was performed with SYBR^®^ Premix Ex TaqTM (Takara, Japan) on a StepOne plus real-time PCR detection system (Applied Biosystems, Foster City, CA, USA). The specific primers (Generay Biotech Co., Shanghai, China) are listed in Table S1. The expression levels of each gene were normalized to glyceraldehyde-3-phosphate dehydrogenase (GAPDH) using the comparative 2^−∆∆*C*t^ method.

### 4.5. Glucose Tolerance Test (GTT)—HOMA-IR

An intraperitoneal glucose tolerance test was performed on SD rats (2 g glucose/kg body weight) after overnight fasting on day 10, 20, 30, 40 and 50 following a high fat-sucrose diet or basal diet feeding. The blood glucose level was examined at 0, 30, 45, 60, 90 and 120 min after glucose injection using a commercial enzymatic assay kit (Biosino Bio-Technology and Science Inc., Beijing, China). Serum insulin was measured using an ELISA kit Cusabio (Cusabio Biotech, Wuhan, China). Insulin resistance (IR) was assayed with homeostasis model assessment (HOMA)-IR, using the following formula: HOMA-IR = (glucose (mmol/L) × insulin (μU/mL))/22.5.

### 4.6. Western Blotting Analysis

Liver tissues were homogenized at 4 °C with an extraction buffer, and the supernatants were used for western blotting analysis, with AKT (1:1000, Cell Signaling Technology, Danvers, MA, USA), pAKT^ser473^ (1:2000, Cell Signaling Technology) and β-actin (1:2000, Sigma-Aldrich, St. Louis, MO, USA) as the primary antibodies and Goat Anti-Rabbit IgG, HRP (1:8000, jackson immunoresearch, West Grove, PA, USA) and Goat Anti-Mouse IgG, HRP (1:4000, abcam) as the secondary antibodies. Antibody expression was viewed by applying enhanced chemiluminescence reagents (Millipore, Billerica, MA, USA). Quantity One was used to quantify the band intensities (Bio-Rad, Hercules, CA, USA).

### 4.7. Lipid and Hepatocellular Oxidative Stress Analysis of the Liver

Lipids extracted from liver were dissolved in isopropanol, and then TG and TC contents in liver were analyzed as described above. The hepatic malondialdehyde (MDA) and superoxide dismutase (SOD) (Nanjing Jiancheng Bioengineering Institute, Nanjing, China) levels were measured following the manufacturer’s protocols.

### 4.8. Statistical Analysis

All results are expressed as the mean ± SEM. Comparisons between groups were evaluated by one-way analysis of variance (ANOVA) and Student’s *t*-test. The statistics were performed using prism Version 5.01 (GraphPad Software, San Diego, California, CA, USA). The data were considered statistically significant when *p* < 0.05.

## Figures and Tables

**Table 1:** Effect of high fat-sucrose diet on body weight, liver index and food intake of Sprague-Dawley (SD) rats (Mean ± Standard Error of Mean (SEM), *n* = 6).

Groups	Body Weight (g)	Liver Index	Food Intake (g/day)
C10	323.75 ± 13.54	0.033 ± 0.003	26 ± 0.3
M10	264.28 ± 11.35 ^###^	0.039 ± 0.003	24 ± 0.8
C20	347.93 ± 9.40	0.031 ± 0.004	27 ± 0.6
M20	284.00 ± 11.37 ^###^	0.041 ± 0.002 ^##^	26 ± 0.8
C30	408.22 ± 18.37	0.028 ± 0.002	25 ± 0.9
M30	283.08 ± 11.80 ^###^	0.045 ± 0.003 ^###^	26 ± 0.8
C40	422.22 ± 10.62	0.032 ± 0.005	25 ± 0.8
M40	279.47 ± 11.99 ^###^	0.044 ± 0.003 ^##^	27 ± 0.4
C50	435.46 ± 18.17	0.033 ± 0.002	24 ± 0.4
M50	267.28 ± 14.51 ^###^	0.043 ± 0.003 ^##^	26 ± 0.6

Liver index: the ratio of liver wet weight and body weight. Compared with the corresponding control group, ^##^ *p* < 0.01, ^###^ *p* < 0.001.

## Supplementary Materials

Supplementary materials can be found at www.mdpi.com/1422-0067/18/8/1681/s1.
