# Supplementary material for: Comprehensive Study of Multiple Stages Progressing to Nonalcoholic Steatohepatitis with Subsequent Fibrosis in SD Rats
Source: Int J Mol Sci. 2017 Aug 18;18(8):1681. doi: 10.3390/ijms18081681 (PMC5578071; doi:10.3390/ijms18081681)
Supplement: Supplementary file 1 [file ijms-18-01681-s001.pdf]

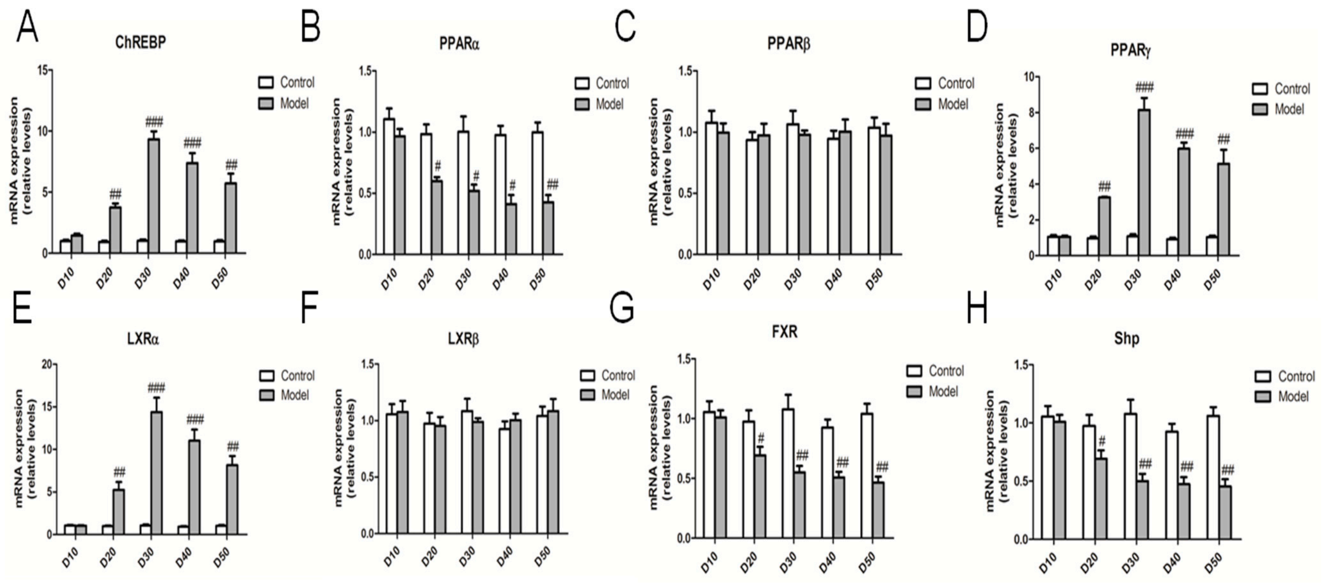

Figure S1. Effect of high fat and high sucrose diet on hepatic lipid metabolism in SD rats. The hepatic lipid metabolism parameters of SD rats induced by high fat and high sucrose diet for 10, 20, 30, 40, 50 days. The mRNA gene expression for ChREBP (A), PPARα (B), PPARβ (C), PPARγ (D), LXRα (E), LXRβ (F), FXR (G), Shp (H). The values are expressed as the means ± SEM (n = 6). Compared with the corresponding control group, # $p < 0.05$, ## $p < 0.01$, ### $p < 0.001$.

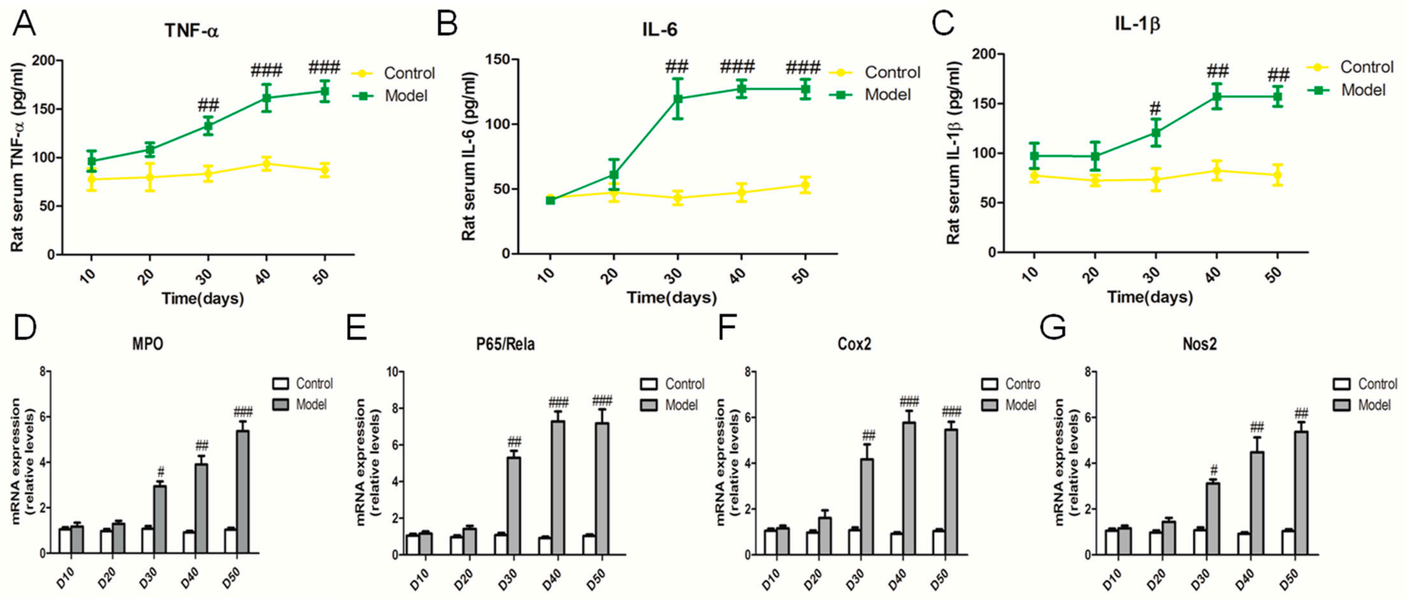

Figure S2. Effect of high fat and high sucrose diet on inflammation factor in SD rats. The system inflammation parameters of SD rats induced by high fat and high sucrose diet for 10, 20, 30, 40, 50 days. The serum inflammation factors for TNF-α (A), IL-6 (B), IL-1β (C), The hepatic inflammation factors for MPO (D), P65/Rela (E), Cox2 (F), Nos2 (G). The values are expressed as the means ± SEM (n = 6). Compared with the corresponding control group, , # $p < 0.05$, ## $p < 0.01$, ### $p < 0.001$.

Table S1. Primers used for quantitative real-time polymerase chain reaction.

Gene	Forward primer (5'-3')	Reverse primer (5'-3')	NCBI GeneBank
PPARγ2	CAGGTTTGGGCGAATG	TTTGGTCAGCGGGAAG	NM_001145366.1
SREBP1c	GGAGCCATGGATTGCACATT	GCTTCCAGAGAGGAGCCCAG	NM_001276708.1
FAS	CAACATTGACGCCAGTTCCG	TTCGAGCCAGTGTCTTCCAC	NM_017332.1
TNF-α	CACCATGAGCACGGAAAGCATGA	CGCCTCACAGAGCAATGACTCCA	NM_012675.3
IL-6	CACTTCACAAGTCGGAGGCT	AGCACACTAGGTTTGCCGAG	NM_012589.2
MCP-1	GATCCCAATGAGTCGGCTGG	ACAGAAGTGCTTGAGGTGGTT	NM_031530.1
TGF-β1	CTTTGTACAACAGCACCCGC	TAGATTGCGTTGTTGCGGTC	NM_021578.2
α-SMA	CCCTGAAGTATCCGATAGAACA	TGCCTGGGTACATGGTAGTG	NM_031004.2
MMP-13	GTGACTCTTGCGGGAATCCT	CAGGCACTCCACATCTTGGT	NM_133530.1
CAT	GAGGCAGTGTACTGCAAGTTCC	GGGACAGTTCACAGGTATCTGC	NM_012520.2
GPx1	TCCACCGTGTATGCCTTCTCC	CCTGCTGTATCTGCGCACTGGA	NM_030826.3
GAPDH	CCTTCATTGACCTCAACTACATGGT	TCATTGTCATACCAGGAAATGAGCT	NM_017008.4
Mlxipl	CGGGGTCGTGTAGACAACAA	CTGACAAGTCCGTGCAGAGT	NM_133552.1
PPARα	GCATGGCTGAGAAGACGCTTG	GGATAGCCTTGGCAAATTCCG	NM_013196.1
PPARβ	AGCAGCCTCTTCCTCAACGAC	AACTTGGGCTCAATGATGTCAC	NM_013141.2
PPARγ	GAAGCCCTTTGGTGACTTTATG	AGGTTGTCTTGGATGTCCTCGA	NM_013124.3
LXRα	GGAGCACGCTACATTTGCCATA	CCTCTTCTTGACGCTTCAGTTTC	NM_031627.2
LXRβ	AGACGCTACAACCACGAGAC	CCGCTTGATCCTCGTGTAGG	NM_031626.1
FXR	GCCCACTGAGAGTGTGTACC	TCCCATCTCTCTGCACTTCC	NM_021745.1
Shp	TGATGGCTCCCAAAACCTCC	AGGAATTCTGCCCTGAAGCA	NM_057133.1
Rela /P65	CATGGATCCCTGCACACCTT	CTCAGCATGGAGAGTTGGCA	NM_199267.2
Cox2	TGTGAAAGGGTGTCCCTTCG	AGTACAACACAGGAATCTTCACA	S67722.1
Nos2	GGTGAGGGGACTGGACTTTTAG	TTGTTGGGCTGGGAATAGCA	NM_012611.3
